# Investigation of the Relationship Between Physical Activity Level, Posture Disorder and Cardiopulmonary System in University Students

**DOI:** 10.1007/s10900-026-01552-3

**Published:** 2026-04-20

**Authors:** Abdurrahim Yıldız, Ece Cömer, Sude Sultan Deniz, Rojbin Ektiren

**Affiliations:** Faculty of Health Sciences, Physiotherapy and Rehabilitation Application and Research Center, Sakarya University of Applied Sciences, Yeni Mah. Alay Cad. No:18 Akyazı, Sakarya, Turkey

**Keywords:** Physical activity, Posture, Students, Sedentary behavior

## Abstract

To examine the relationship between physical activity level, postural disorders and cardiopulmonary functions in university students. Regular physical activity is known to support postural balance and improve the functions of the cardiopulmonary system through its positive effects on the musculoskeletal system. In this context, considering the prevalence of sedentary lifestyle in university students, the effects of physical activity on these systems were investigated. The study was conducted with 164 volunteer students between the ages of 18–25 years studying at Sakarya University of Applied Sciences. The International Physical Activity Questionnaire (IPAQ) was used to assess the physical activity levels of the participants, the SF-36 Quality of Life Scale was used to assess quality of life, the 6 min Walk Test (6MWT) was used to measure cardiopulmonary capacity, and the Posture Analysis Method was used to assess posture disorders. The findings of the study showed that low physical activity level was associated with postural imbalances and decreased cardiopulmonary capacity. It was observed that as the physical activity level increased, postural disorders decreased and cardiopulmonary functions improved. A positive correlation was found between physical activity levels and physical function, energy/vitality and general health subscales of SF-36 scale. In addition, 6MWT performance showed better results in individuals with higher physical activity levels. It has been demonstrated that regular physical activity supports postural balance and improves the functions of the cardiopulmonary system in university students. It is clear that interventions to increase the level of physical activity will provide significant benefits on students’ postural and cardiopulmonary health in this period when sedentary lifestyle is common. It is recommended to develop programs that encourage physical activity in universities.

## Introduction

 Physical activity is a globally used term describing body movements by using energy. In daily life, activities such as using muscles and joints, providing energy consumption, increasing respiratory rate and cardiac output constitute physical activity [1]. Regular physical activity has positive effects on health. It is an important part of energy expenditure. In current studies, it has been proven that physical activity has positive effects on physical fitness and health in people who have made physical activity a habit [2]. This process also has benefits in maintaining body alignment. Posture is defined as the sitting or standing position of a person’s body and is divided into two. One of them is known as good posture and the other is known as bad posture [3]. Situations such as sociodemographic and economic status, nutrition, social life, work and occupations, emotional state, sports habits directly or indirectly affect the posture of the person [4]. Exercise is the most broadcast physiological stress of the body and has great effects on the cardiopulmonary system. Therefore, exercise can be considered as the most practical functional test of the cardiac system [5]. The cardiovascular system responds to acute exercise by a series of regulations that ensure that active muscles receive blood supply appropriate to their metabolic needs, that heat generated by the muscles is dissipated, and that blood flow to the brain and heart is maintained. This response, together with a series of local metabolic changes, results in a large redistribution of cardiac output [6].

The relationship between physical activity level, postural disorders and cardiopulmonary system in university students shows an important interaction in terms of health and performance. Physical activity supports postural balance by creating positive effects on the musculoskeletal system and helps to prevent postural disorders [7]. In particular, low levels of physical activity can lead to postural disorders due to poor muscle tone and lack of flexibility. Postural disorders, in turn, can adversely affect the functioning of the rib cage and diaphragm, putting pressure on the cardiopulmonary system. This can lead to reduced respiratory function, reduced lung capacity and poor cardiovascular endurance [8]. On the other hand, regular physical activity strengthens the cardiopulmonary system, supports the respiratory muscles and increases oxygen utilisation, thus minimising the negative effects caused by postural disorders [9]. The prevalence of sedentary lifestyle in university students may cause these problems to be seen more frequently and the importance of regular exercise should be emphasised. When we look at the literature, many studies have been conducted on physical activity, but it is seen that the studies examining the relationship between physical activity and postural disorders and cardiopulmonary functions in university students are limited. Therefore, in our study, we aimed to investigate the relationship between physical activity level and quality of life, postural disorders and cardiopulmonary functions in university students.

## Method

### Type of Research

This study was planned as a cross-sectional study.

### Population and Sample of the Study

The study was conducted between March-June 2024 at Sakarya University of Applied Sciences, Akyazı Faculty of Health Sciences and Akyazı Vocational School of Health. The inclusion criteria were; volunteering to participate in the study, being between the ages of 18–25, being a university student. Exclusion Criteria; having a neurological or orthopaedic disease, having cardiac and pulmonary problems, having visual and auditory problems were not included in the study.

The number of students to be sampled was calculated with the G*Power 3.1.9.2 programme. It was planned to include 163 students in the sample by accepting the effect level as medium (0.30), power level as 99% and significance level as 0.05.

### Ethical Methodology of the Research

Permission for the study was obtained from the Ethics Committee of Sakarya University of Applied Sciences on 20.02.2024 with the number E.117,607.

### Data Collection

The purpose of the questionnaires applied to the individuals in each group and the protocol of the application were explained in detail before starting the study to all participants. ‘Informed Voluntary Consent’ was obtained from the university students who volunteered to participate in the study by explaining the purpose of the study, that all personal information would remain confidential and that the research data would not be shared with anyone. The demographic information of the participants (gender, age, weight and height) was collected in the form of question-answer in the questionnaire. The data in the study were obtained with the International Physical Activity Questionnaire (IPAQ), SF36 Quality of Life Questionnaire, 6-minute walk test (6MWT), and Posture Analysis Method.

### Statistical Analysis

SPSS for Windows Version 27 package program was used in the evaluation and statistical analysis of the data obtained in the study. Descriptive statistics were shown as number and % for variables determined by counting and mean ± standard deviation for variables determined by measurement. Paired t test was used to compare the values before and after the test. In addition, the relationship between the parameters was evaluated using Spearson correlation test. *P* < 0.05 level was accepted as an indicator of significant difference.

#### Assessment of Physical Activity

The IPAQ scale developed by Craig et al. to examine the physical activity of participants aged between 15 and 65 years measures activities such as vigorous intensity physical activity, moderate intensity activity, walking and sitting. When evaluating the activities, each activity in the questionnaire should be performed for at least 10 min in the last 7 days and when the activity is started. Minutes, days and metabolic equivalent (MET) are multiplied to obtain a score as ‘MET-minutes/week’. As a result of the survey, MET-min/week is below 600 in people who are not physically active. In people with low physical activity level, the MET-min/week product is between 600 and 3000, while the physical activity level that is sufficient for health takes the value of 3000 MET-min/week [10].

#### Assessment of Cardiopulmonary Capacity

Cardiopulmonary capacity of the person can be evaluated with 6MWT. 6MWT expresses the distance in metres that the person takes in 6 min. It is a test developed in 1963 by Balke to measure functional capacity. At the same time, the parameter tried to measure is maximum oxygen consumption. The normal value of the distance measured at the end of the test is expected to be between 400 and 700 m. The individual is rested 15 min before the test and then the distance walked for 6 min at max walking speed on a 30-metre track is measured and recorded. Before starting the test, the patient is informed and the commands to be followed are explained. Blood pressure, pulse rate, fatigue rate and SPO2 values are measured and recorded at the beginning and end of the test. Individuals should have eaten a light meal at least one hour before the test and should not have performed heavy physical activity within 2 h before the test.

#### Assessment of Quality of Life

SF-36 Quality of Life Scale is a scale developed by Ware and Sherbourne. Although it is generic among quality of life scales, it allows a comprehensive assessment. Although its validity and reliability was performed by Koçyiğit et al. in individuals with physical illness in Turkey, it is a widely used scale. In addition to being short-form, it includes questions filled in by the individuals themselves and answered about their health status in the last 4 weeks. It is a questionnaire consisting of 36 items measuring health-related quality of life. It has 8 subheadings. In the SF-36 quality of life questionnaire, all sections are evaluated by scoring between 0 and 100. While 0 points indicate negative changes in health, 100 points indicate that there is no problem in the health of the individual. When the scale is evaluated, each section is scored within itself. It is not possible to calculate a total score. While some parts of the scale are evaluated with Likert type, some parts are evaluated with yes/no questions.

#### Posture Assessment

In this assessment system, posture changes that may occur in 10 different parts of the body are scored observationally. Posture is the optimal position of all body parts. Posture analysis is performed while standing in anatomical position. In the posture analysis, observations are evaluated from two angles, lateral (side) and posterior. The aim is to determine good or bad posture. The scoring of the test is 10 points if the person has proper posture, 5 points if there is a moderate disorder, and 0 points if there is a serious disorder. The result of the analysis takes a value between 0 and 100. As you go from 100 to 0, the increase in posture disorder is observed.

#### Assessment of Cough Strength

Peak Expiratory Flow (PEF) measurement to evaluate cough force (PCF) is performed with standard portable PEF meters which are practical to use. At the beginning of the PCF meter measurement, the needle is brought to zero, the mouthpiece part is grasped with the lips, a deep breath is taken, the breath taken is exhaled suddenly and quickly, and the value shown by the needle is measured and recorded. The process is done 3 times and the highest value is taken as the base.

## Results

**Fig. 1:**
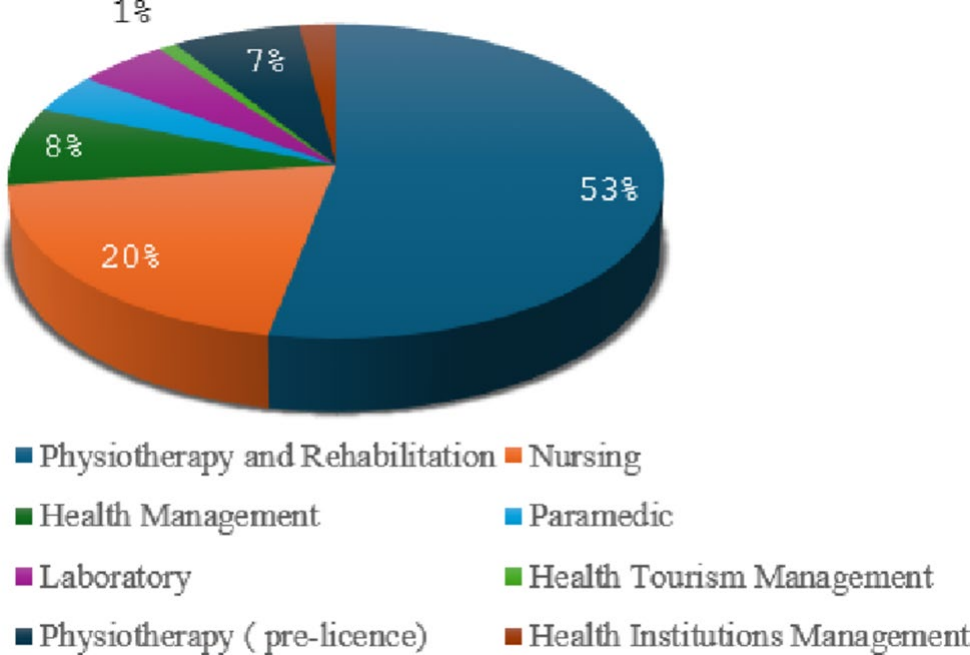
Departments studied by the participants and their percentages

The study was planned to investigate the effect of physical activity level on quality of life, cardiopulmonary system and posture in university students. For this purpose, 126 female (76.8%), 38 male (23.2%) and 164 volunteer university students who were continuing their education at Sakarya University of Applied Sciences were evaluated within the scope of our study. The mean age of the individuals participating in the study was 21.04 years, the mean height was 165.88 and the mean body mass index was 37.17. Of the 164 individuals who participated in the study, 131 undergraduate (79.9%) and 33 associate degree (20.1%) participated. The departments of the individuals were shown in Fig. 1.

Table 1 shows the distribution of the mean scores of the sub-dimensions of the quality of life scale of university students. The mean scores of the university students were 86.59 ± 18.47 for physical function, 75.56 ± 30.65 for role limitation related to physical function, 68.45 ± 20.03 for bodily pain, 59.45 ± 16.51 for general health, 56.34 ± 17.83 for mental health, 58.53 ± 41.14 for role limitation related to emotional function, 69.56 ± 21.07 for social function, and 51.71 ± 18.24 for vitality/energy.

**Table 1:** Mean scores of SF-36 scale sub-dimensions of university students

Quality of Life Subscales	Mean ± SD
Physical Function	86.59 ± 18.47
Role limitation due to physical function	75.56 ± 30.65
Physical Pain	68.45 ± 20.03
General Health	59.45 ± 16.51
Mental Health	56.34 ± 17.83
Role Restriction Due to Emotional Functioning	58.53 ± 41.14
Social Function	69.56 ± 21.07
Vitality/Energy	51.71 ± 18.24

In Table 2, the mean scores of SPO2, systolic blood pressure, diastolic blood pressure, pulse and fatigue values of university students before and after 6MWT were compared. According to this, it was seen that the values of large blood pressure, small blood pressure, pulse and fatigue values measured after 6MWT were statistically significant compared to before (*p* < 0.001). There was no statistically significant difference in SPO2 values before and after 6MWT.

**Table 2:** Comparison of the tests of individuals before and after 6MWT

	Before test	After test	*p*
	Mean ± SD	Mean ± SD	
SPO2	97.57 ± 2.46	98.47 ± 1.07	0.401
Diastolic Blood Pressure	68.57 ± 9.39	71.93 ± 10.92	p < 0.001*
Systolic Blood Pressure	117.21 ± 12.54	122.87 ± 12.79	p < 0.001*
Pulse	84.97 ± 12.48	95.75 ± 12.48	p < 0.001*
Fatigue	1.43 ± 0.64	1.56 ± 0.69	p < 0.001*

*Paired t test, *p* < 0.05

Lateral flexion of the head showed moderate lateral flexion in 59.8% (*n* = 98) and severe lateral flexion in 1.2% (*n* = 2). When the shoulder was evaluated, moderate scapular inequality was observed in 29.3% (*n* = 48) and no scapular inequality was observed in 70.7% (*n* = 116). When the spine was evaluated, moderate curvature was observed in 10.4% (*n* = 17), while 89.6% (*n* = 147) had no problem in the spine. As a result of hip evaluation, moderate SIPS inequality was observed in 7.3% (*n* = 12), while no inequality was observed in 92.7% (*n* = 152). As a result of ankle evaluation, 12.8% (*n* = 21) had moderate eversion problems, while 0.6% (*n* = 1) had severe eversion problems. When the neck was evaluated, moderate anterior tilt was observed in 26.8% (*n* = 44), while 72.6% (*n* = 119) had severe anterior tilt. When kyphosis was evaluated, moderate kyphosis was observed in 8.5% (*n* = 14) and severe kyphosis was found in 0.6% (*n* = 1). In the evaluation of the lumbar region, 4.9% (*n* = 8) had moderate lordosis, while 0.6% (*n* = 1) had advanced lordosis. As a result of the analysis, 12.2% (*n* = 20) had moderate posterior pelvic tilt, 0.6% (*n* = 1) had severe posterior pelvic tilt, 9.8% (*n* = 16) had moderate anterior pelvic tilt and 0.6% (*n* = 1) had severe pelvic tilt (see Table 3).

**Table 3:** Posture analysis and evaluation according to body parts

Body Parts		0	5	10
Head	n	2	98	64
	(%)	%1.2	%59.8	%39
Shoulder	n	0	48	116
	(%)	%0	%29.3	%70.7
Spine	n	0	17	147
	(%)	%0	%10.4	%89.6
Hip	n	0	12	152
	(%)	%0	%7.3	%92.7
Ankle	n	1	21	142
	(%)	%0.6	%12.8	%86.6
Neck	n	1	44	119
	(%)	%0.6	%26.8	%72.6
Back	n	1	14	149
	(%)	%0.6	%8.5	%90.9
Body	n	1	8	155
	(%)	%0.6	%4.9	%94.5
Abdomen	n	1	20	143
	(%)	%0.6	%12.2	%87.2
Lower back	n	1	10	147
	(%)	%0.6	%9.8	%88.6

**Table 4:** Correlation of SF-36, 6MWT, PCF, IPAQ and BMI values

Quality of Life Subscales	6MWT		PCF		IPAQ		BMI	
	*r*	*p*	*r*	*p*	*r*	*p*	*r*	*p*
Physical Function	0.198	0.011*	0.056	0.475	0.119	0.128	0.027	0.733
Role limitation due to physical function	0.097	0.218	0.188	0.016*	0.077	< 0.001*	0.436	< 0.001*
Physical Pain	0.165	0.034*	0.154	0.048*	0.014	0.857	0.027	0.734
General Health	0.240	0.002*	0.253	< 0.001*	0.153	0.051	0.047	0.552
Mental Health	0.092	0.242	0.030	0.699	0.043	0.584	0.806	< 0.001*
Role Restriction Due to Emotional Functioning	0.218	0.005*	0.111	0.143	0.008	0.917	0.042	0.591
Social Function	0.036	0.644	0.131	0.094	0.006	0.939	0.015	0.853
Vitality/Energy	0.276	< 0.001*	0.056	0.477	0.114	0.145	0.064	0.417
Physical Function	0.755	< 0.001*	0.514	< 0.001*	0.085	0.280	0.161	< 0.001*

BMI: body mass index, PCF: peak cough flow rate, IPAQ: international physical activity questionnaire, 6MWT: Six Minute Walking Test

*Spearman corelation test, *p* < 0.05

The correlation between 6MWT and the sub-parameters of SF36 was found to be positive between 6MWT and SF36-physical function sub-parameter (*p* = 0.011), positive with physical pain sub-parameter (*p* = 0.034), positive with general health sub-parameter (*p* = 0. 002), positive correlation with vitality/energy sub-parameter (*p* < 0.001), positive correlation with role limitation due to emotional function (*p* = 0.005), positive correlation with health change sub-parameter (*p* < 0.001). When we looked at the correlation between the sub-parameters of Sf36 and PPSD; there was a significant positive correlation (*p* = 0.016) between 6MWT and SF36- role limitation due to physical functioning sub-parameter, a significant positive correlation (*p* = 0.048) with physical pain sub-parameter, a significant positive correlation (*p* < 0.001) with general health sub-parameter, and a significant positive correlation (*p* < 0.001) with health change sub-parameter. When we looked at the correlation between IPAQ and the sub-parameters of Sf36, it was seen that there was a significant positive correlation (*p* < 0.001) between IPAQ and the sub-parameter of SF36-role limitation due to physical function. When we looked at the correlation between BMI and the sub-parameters of Sf36; there was a significant positive correlation (*p* < 0.001) between BMI and the sub-parameter of role limitation related to physical functioning, a significant positive correlation (*p* < 0.001) with the sub-parameter of mental health, and a significant positive correlation (*p* < 0.001) with the sub-parameter of health change (Table 4).

It was observed that there was a positive correlation between the total score of physical activity levels assessed with IPAQ and 6MWT distance (*p* = 0.017), positive correlation with PCF (*p* = 0.024), and positive correlation with BMI (*p* = 0.039). In addition, there was a positive correlation with IPAQ vigorous physical activity score and 6MWT walking distance. No significant correlation was found between IPAQ subscores and 6MWT distance, PCF and BMI. In addition, there was a positive correlation between PCF and BMI (*r* = 0.169, *p* = 0.030) and between PCF and 6MWT distance (*r* = 0.262, *p* = 0.001) (see Table 5).

**Table 5:** Correlation between UFAA scores and 6DYT, PCF and BMI

	6MWT		PCF		BMI	
	*r*	*p*	*r*	*p*	*r*	*p*
IPAQ	0.186	0.017*	0.177	0.024*	0.161	0.039*
IPAQ -severe PA	0.304	< 0.001*	0.138	0.079	0.142	0.070
IPAQ -moderate PA	0.068	0.390	0.079	0.312	0.087	0.270
IPAQ -walking	0.096	0.220	0.111	0.158	0.092	0.243

BMI: body mass index, PCF: peak cough flow rate, IPAQ: international physical activity questionnaire, 6MWT: Six Minute Walking Test

*Spearman corelation test, *p* < 0.05

## Discussion

Our study was conducted with 164 students studying in 8 different departments of the Faculty of Health Sciences and School of Health Vocational School within Sakarya University of Applied Sciences in order to investigate the relationship between physical activity level, quality of life, posture and cardiopulmonary functions in university students. In our study, the relationship between physical activity levels of university students on quality of life, posture and cardiopulmonary system was determined and the factors affecting this relationship were shown. In our study, the relationship between the participants’ SF-36 values ​​and 6MWT, PCF, BMI values ​​was examined. As a result of the study, a significant difference was found between SF-36 and 6MWT, PCF, BMI.

Based on the findings of our study analysis, it was seen that the participation of university students in physical activities was at a low level, the rate of people who did not do severe physical activity was 65.2% (*n* = 107), the rate of people who did not do moderate physical activity was 56.7% (*n* = 93), and the average sitting rate per day varied between 6 and 8 h. No correlation was observed between IPAQ and quality of life. These results show that there is a lack of physical activity in university students. Quality of life, which is one of the important goals that societies aim for today, covers all areas of life and is affected by all areas of life. Physical activities performed in daily life play a great role on quality of life [11]. As the physical activity level of individuals increases, their quality of life also increases to a great extent. In a study conducted by Bize et al. on exercise and quality of life, it was found that there was a positive relationship between the level of physical activity reported by the individual and health-related quality of life [12]. When we look at our study results, a significant difference was found between the SF36 sub-parameter of role limitation due to physical function and the IPAQ total score. This led us to conclude that the limitation in daily life due to physical activity and lack of physical activity would decrease depending on the increase in activity level. In studies conducted in different populations, physical activity level was found to be effective in 6MWT performance. In a study, a significant correlation was found between daily walking period and 6MWT in 65-year-old people [13]. In another study examining the difference in body weights in children, it was found that children with normal body weight walked more distance in the 6MWT than children with excess body weight [14]. In our study, a positive significant correlation was found between IPAQ and 6MWT. Some cross-sectional studies have also found that physical activity has an effect on physical fitness depending on its intensity.

The findings of our study emphasise the negative effects of low levels of physical activity on muscle tone and posture and the pressure on the cardiopulmonary system. On the other hand, regular physical activity has been shown to reduce these negative effects and contribute to the maintenance of postural balance and more efficient functioning of the respiratory and circulatory systems. In support of our study, Karabıçak and Baltacı investigated the effect of physical activity level on posture in adolescents and found no significant relationship between physical activity and posture [15]. In a study involving 366 university students, no significant difference was found in posture disorders with varying levels of physical activity, but higher activity levels positively affected depression and sleep quality [16]. Conversely, research from Anhui Polytechnic University has shown that appropriate physical exercise is a positive factor in preventing poor posture, suggesting that lifestyle habits, including physical activity, play a crucial role in posture health [17, 18]. Interventions aimed at increasing physical activity not only improve mental health, but may also indirectly benefit posture through improved general well-being [16, 18]. The targeted approach to physical education, including specific exercises, has shown effectiveness in correcting posture disorders among students [18]. Since all of the students participating in our study were from health departments, 53% were from physiotherapy and rehabilitation department and 7% were physiotherapy associate degree students, we think that postural awareness is high in people. We believe that this reduces the rate of postural disorder.

Variables such as BMI have been associated with spirometric measurements and/or cardiopulmonary variables such as VO2max in many studies [19, 20]. The relationship between BMI and PCF variables was analysed in our study. The mean BMI of the individuals who participated in our study was 22.38, which was within the normal range. According to the analyses in our study, there was a significant positive correlation between BMI and PCF. The study conducted by Lazarus et al. was not parallel to our study because the study population was different. The studies generally showed a negative correlation because they were conducted with obese individuals, whereas a positive correlation was found in studies with BMI values within normal limits [21]. The tendency towards sedentary life with the advancement of technology causes limitations in thoracic cage mobility, lung capacity and respiratory functions in individuals. Therefore, even healthy individuals with inactive lifestyles may experience respiratory problems in the future [22]. A positive correlation was found between the SF36-subparameter vitality/energy and 6MWT. Inadequate cardiac capacity causes an increase in fatigue levels and shortening of walking distance. In our study, no significant difference was found between the pre-test and post-test values of SPO2. The reason for this was that the study population consisted of healthy individuals and no difference was observed in SPO2 values.

### Limitations

Our study has some limitations. The fact that the university students who constitute the study sample in the data collection process of the study consisted only of the students of the Faculty of Health Sciences and Health Vocational School cannot provide information about the departments other than the health department. We are of the opinion that the studies to be conducted with a larger sample group will contribute more to the literature and more accurate results will be obtained.

## Conclusion

Our study investigated the relationship between physical activity level and posture disorders and cardiopulmonary functions in university students. According to the results obtained, regular physical activity was found to support postural balance and improve the functions of the cardiopulmonary system. In particular, it was found that students with high physical activity levels had fewer problems in terms of postural disorders and had better cardiovascular endurance.

The findings of the study emphasise the negative effects of low levels of physical activity on muscle tone and posture and the pressure this puts on the cardiopulmonary system. On the other hand, regular physical activity was found to reduce these negative effects and contribute to the maintenance of postural balance and more efficient functioning of the respiratory and circulatory systems.

In conclusion, physical activity has an important effect on the health of university students. Especially in this period when sedentary lifestyle is common, it is clear that interventions to increase physical activity will provide great benefits in terms of postural and cardiopulmonary health. In this context, it is recommended to develop physical activity promotion programmes for students in universities.

## Data Availability

The datasets generated and/or analyzed during the current study are available from the corresponding author on reasonable request.
